# Tumor Microenvironment Targeted Nanotherapy

**DOI:** 10.3389/fphar.2018.01230

**Published:** 2018-10-31

**Authors:** Clara Fernandes, Divya Suares, Mayur C Yergeri

**Affiliations:** Shobhaben Pratapbhai Patel School of Pharmacy and Technology Management, SVKM's Narsee Monjee Institute of Management Studies - NMIMS, Mumbai, India

**Keywords:** tumor microenviroment, cancer, nano therapy, nano carrier, resistance

## Abstract

Recent developments in nanotechnology have brought new approaches to cancer diagnosis and therapy. While enhanced permeability and retention effect promotes nano-chemotherapeutics extravasation, the abnormal tumor vasculature, high interstitial pressure and dense stroma structure limit homogeneous intratumoral distribution of nano-chemotherapeutics and compromise their imaging and therapeutic effect. Moreover, heterogeneous distribution of nano-chemotherapeutics in non-tumor-stroma cells damages the non-tumor cells, and interferes with tumor-stroma crosstalk. This can lead not only to inhibition of tumor progression, but can also paradoxically induce acquired resistance and facilitate tumor cell proliferation and metastasis. Overall, the tumor microenvironment plays a vital role in regulating nano-chemotherapeutics distribution and their biological effects. In this review, the barriers in tumor microenvironment, its consequential effects on nano-chemotherapeutics, considerations to improve nano-chemotherapeutics delivery and combinatory strategies to overcome acquired resistance induced by tumor microenvironment have been summarized. The various strategies viz., nanotechnology based approach as well as ligand-mediated, redox-responsive, and enzyme-mediated based combinatorial nanoapproaches have been discussed in this review.

## Introduction

Worldwide, tackling cancer still remains a daunting task for clinicians and researchers. Ferlay et al. (2015) have reported that among the different types of cancers, lung cancer is prominently associated with highest mortality rate followed by liver and stomach cancer. In recent times, there has been increased incidences of patients afflicted with breast and colorectal cancers. By the year 2025, it is estimated that, globally, there will be a surge in the number of cancer cases (>20 million annually) (Zugazagoitia et al., 2016). This alarming statistics has compelled the researchers across the globe to expedite the research for newer and potent molecules to overcome the acquired resistance and eradicate the cancerous cells from the biological milieu. However, the complexity of the disease, demands exhaustive efforts to design chemotherapeutics for curbing tumor growth (Raavé et al., 2018).

Nonetheless, these efforts have been translated into cancer molecules capable of combating the cancer progression, albeit in preclinical setting. Their implementation in the clinical setting is yet fraught with non-specificity resulting in undesirable side effects (Dai et al., 2016). Another debilitating issue plaguing the chemotherapeutic arena is the development of acquired resistance which is often a distressing fact after the initial responsive period for both individual and combinational cancer therapy (Cree and Charlton, 2017). Compelling clinical findings incriminate the presence of malignant and metastatic components in tumor microenvironment to be an underlying mechanism of tumor resistance to chemotherapy (Cheng et al., 2016).

**Figure 1:**
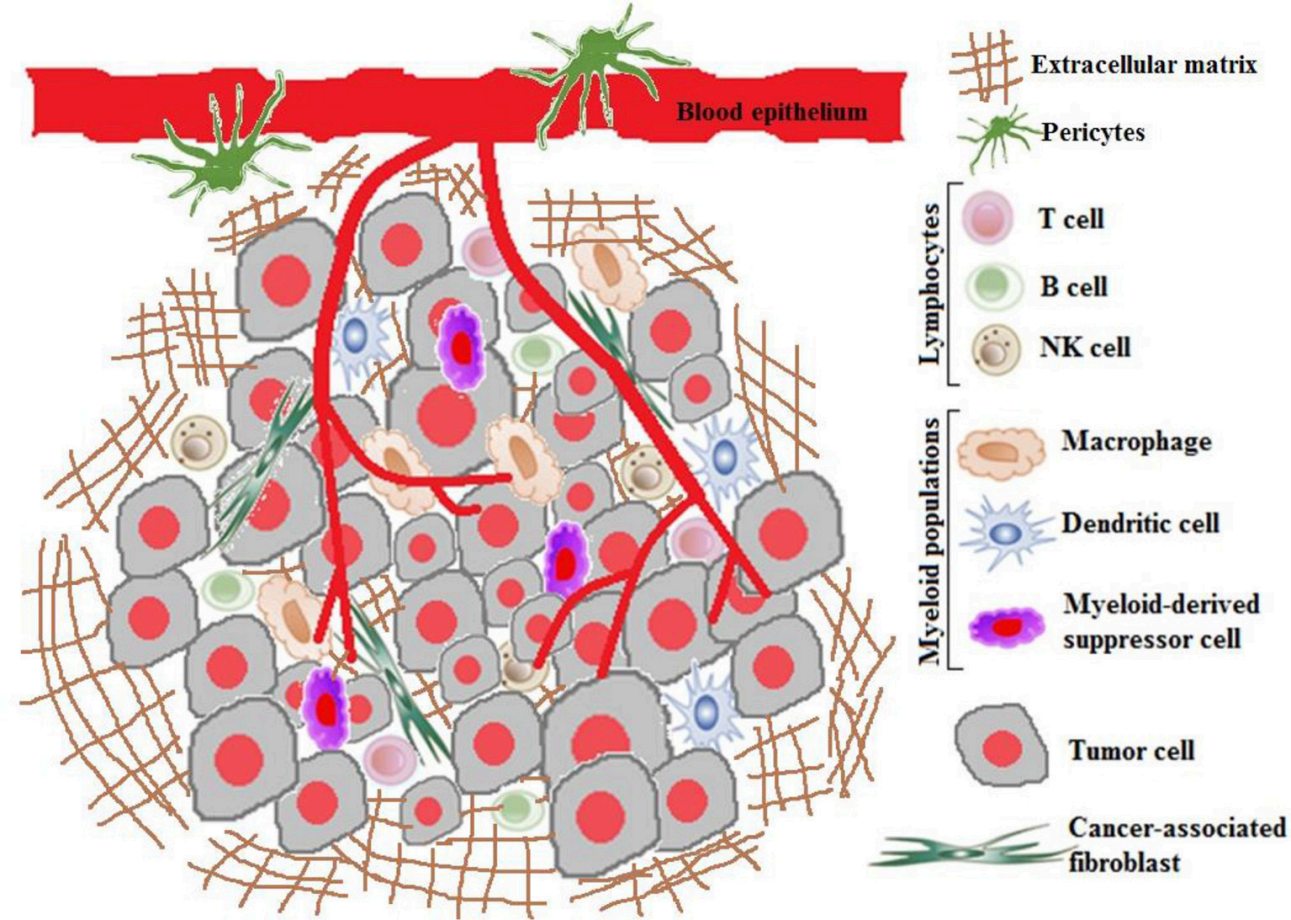
Tumor microenvironment.

Due to complexity of tumor microenvironment (Figure 1), the conventional drug delivery system fails to deliver the chemotherapeutics in effective concentration for cancer cell kill and is associated with debilitating side effects. This has prompted to exploit the alternative nanoparticulate strategy to achieve tumor specificity, if possible, improve therapeutic index and the pharmacokinetic profile of chemotherapeutic agents (Danhier, 2016). By virtue of enhanced permeability and retention (EPR effect), passive diffusion has been found to enable tumor localization of nano-chemotherapeutics. With the limited understanding of tumor microenvironment and initial success accrued exploiting EPR effect; the earlier research was primarily focused on designing stable long-circulating nanocarriers to enable superior drug localization with minimal loss of drugs in systemic circulation. To-date, these efforts have translated into commercialization of first generation FDA-approved nano-chemotherapeutics; liposomal formulation of doxorubicin (DOX) (Doxil® or Caelex®), daunorubicin (DaunoXome®) and albumin-bound paclitaxel (PTX) (Abraxane®) (Overchuk and Zheng, 2017). However, clinically these formulations have been found to be moderately successful due to inadequate tumoral delivery of the nano-chemotherapeutics (Primeau et al., 2005; Kyle et al., 2007). The probable reasons for this discrepancy can be ascribed to the confinement of nano-chemotherapeutics to highly perfused regions, often depriving the low perfused or avascular tumor regions. This inhomogeneity in tumoral distribution has shown to contribute to sub-optimal therapeutic efficacy, acquired resistance, tumor recurrences and hence, necessitates the need for high drug dosing. Consequently, leading to undesirable adverse or toxic effects (Waite and Roth, 2012; Stapleton et al., 2015).

For tumoral uptake, the nano-chemotherapeutics rely on the tumor vasculature wherein they are extravasted into the tumor interstitium. However, within the tumor microenvironment, the localization of nano-chemotherapeutics may also be obstructed by the high interstitial fluid pressure, altered extracellular matrix (ECM) structure, increased cell division and impaired lympathic drainage (Wong et al., 2011; Ozcelikkale et al., 2013). Thus, there is a dearth in understanding of the complex parameters governing these transport processes and localization in tumor are posing huge bottlenecks for designing an effective nano-strategy for eradicating tumor. In this perspective, this review focuses on understanding the barriers and opportunities proffered by tumor microenvironment and summarizes the diverse strategies to modulate tumor microenvironment for enhanced delivery of nano-chemotherapeutics to overcome acquired resistance.

## Understanding the challenges and opportunities presented by tumor microenvironment to nano-chemotherapeutics

**Figure 2:**
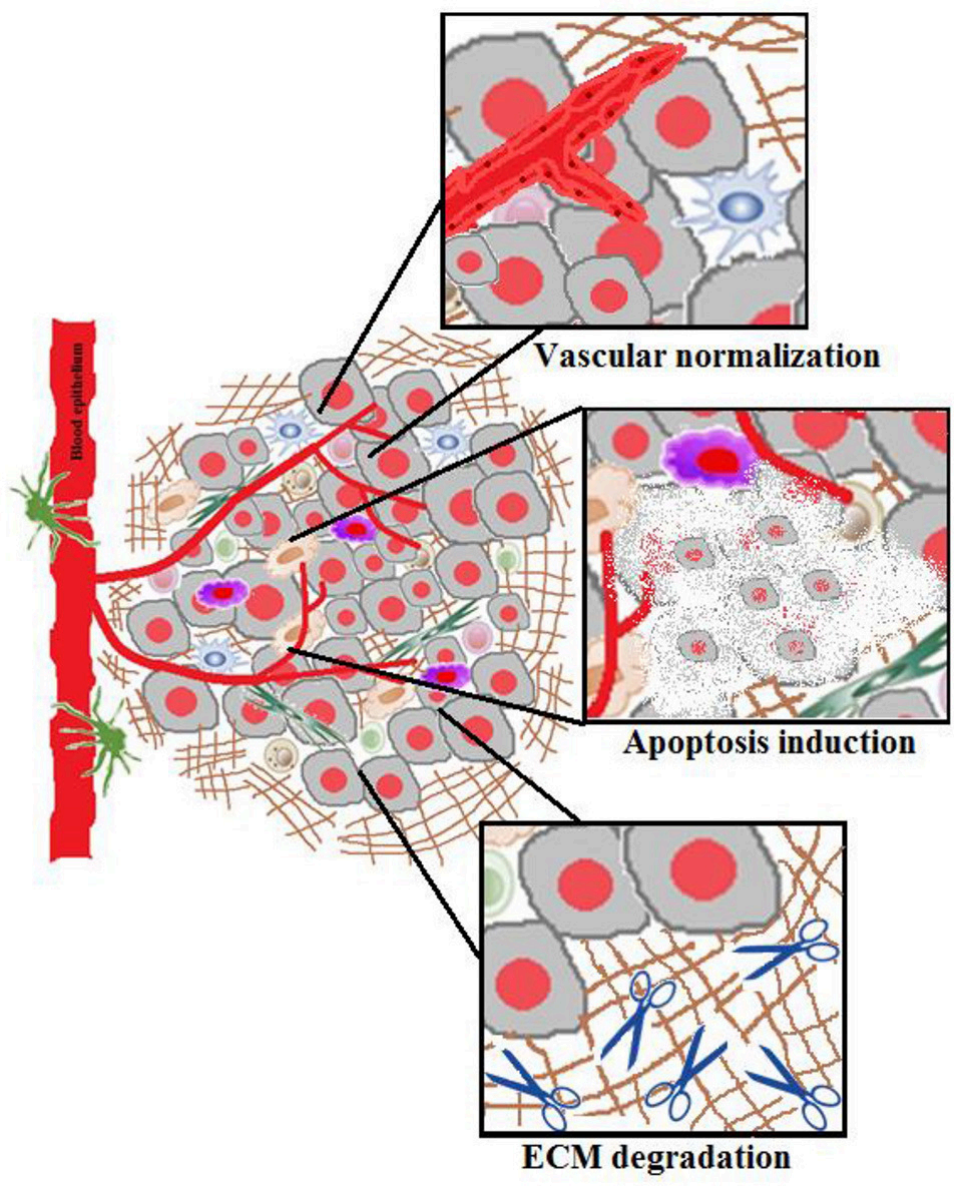
Tumor microenvironment priming.

Mounting evidences give an insight about the crucial role of tumor microenvironment in controlling the abnormal tissue growth, tumor progression, development of localized resistance to chemotherapeutics and metastasis. Overall, tumor microenvironment plays a pivotal role in the therapeutic outcome of the chemotherapeutics in clinical oncology. Thus, it is imperative to have an understanding of the tumor biology for designing effective therapeutic interventions to overcome acquired drug resistance, abrogate tumor progression, and prevent metastasis (Chen et al., 2015). In this section, we recapitulate the hallmarks of the malignant tumor microenvironment which have been targeted for effective anticancer therapy and the challenges for nanoparticulate drug delivery. Broadly, the targeting strategies employed focus on; (i) Priming of tumor microenvironment to facilitate better uptake of nano-chemotherapeutics (Figure 2) and (ii) Tumor targeting of nanocarriers by using suitable approaches designed on the specific expression of receptors, enzymes or modulation of tumor microenvironment.

Considering these stratagems, the section Understanding the Challenges and Opportunities Presented by Tumor Microenvironment to Nano-Chemotherapeutics will provide insight about the tumor priming strategies and section Types of Nanocarriers will discuss the widely known approaches to target nano-chemotherapeutics.

### Tumor-associated vasculature

Generally, tumor-associated vasculature is a key target exploited to achieve localization of the anti-angiogenic chemotherapeutics for suppression of tumor growth. The underlying rationale for anti-angiogenesis, is that an unhindered tumor growth essentially requires independent and unperturbed blood supply. In general, it is assumed that for solid tumors to grow beyond a size of 1–2 mm, a steady supply of oxygen and nutrients is a pre-requisite. Hence, by obstructing the blood supply, tumor regression can be induced *in vivo*. Several mechanisms have been reported (Chen and Cai, 2014) to contribute to tumor-associated vasculature, namely;

i. Sprouting angiogenesis, a dynamic and complex process characterized by formation of new blood vessels arising due to proliferation of endothelial cells of pre-existing capillaries.
ii. Vasculogenesis, a type of “back-up” pathway predominant on inhibition of angiogenesis, wherein, *de novo* capillaries are formed from circulating endothelial progenitor cells (Brown, 2014).
iii. Intussusceptive microvascular growth, another variant of angiogenesis, wherein interstitial tissue pillars (invagination of capillary walls) are inserted into pre-existing capillary resulting in splitting of initial new capillary into two new capillaries. It is considered to be a faster process compared to sprouting angiogenesis and characterized by non-leaky capillaries (De Spiegelaere et al., 2012; Ribatti and Djonov, 2012).
iv. Vessel co-option, a characteristic of aggressive and non-angiogenic tumors, exploits the pre-existing capillaries of the surrounding host tissue. Hence, is a major contributor to resistance to anti-angiogenic therapy and metastasis (Donnem et al., 2013; Bridgeman et al., 2017).
v. Vasculogenic mimicry, an alternate pseudo-vascular channel comprising of predominantly differentiated tumor cells for ensuring blood supply. These channels were discovered initially in highly aggressive melanoma cells. However, in recent times, they have also been reported in other malignant tumors, to name a few, lung cancer, ovarian cancer, breast cancer (Angara et al., 2017; Shen et al., 2017).

The onset of angiogenesis widely known as angiogenic switch is induced by plethora of pro- and anti-angiogenic factors. Most widely known and exploited factors comprise of vascular endothelial growth factor (VEGF), fibroblast growth factor (FGF), platelet-derived growth factor (PDGF), angiopoietin, hypoxia-inducible factor-1 (HIF-1), and transforming growth factor (TGF) which have shown to interact with receptors expressed in the endothelial cells (Carmeliet, 2003; Gacche and Meshram, 2013).

Unlike normal blood vessels which are governed by co-ordinated dynamics of pro- and anti-angiogenic factors, the rapid growing tumor microvasculature are found to be abnormally fragile, irregularly shaped, dilated, tortuous, highly permeable with increased geometric resistance (Geevarghese and Herman, 2014). This abnormality renders the tumor vascular network disorganized and tortuous with a tendency of exclusion of downstream vessels from blood supply. Thus, resulting in discrete hypo-perfused areas or necrotic areas within tumor tissue (Stylianopoulos et al., 2018). Further, the heterogeneous nature of the vascular network, non-laminar blood flow and leaky nature, often result in variability in blood distribution across tumor tissues i.e., showing regions of turbulent or static blood flow. An outcome of this is; (i) Poor accessibility of chemotherapeutics or immune cells present in the bloodstream to poorly perfused tumor regions, (ii) Exacerbation of hypoxic conditions and extracellular acidic pH in tumor, and (iii) Increased interstitial fluid pressure (Jain, 2013; Belli et al., 2018).

#### Tumor pericytes

Although associated with tumor vasculature, in recent times, pericytes, a subtype of mural cells (other types include vascular smooth muscle cells) have garnered attention for their role in tumor microenvironment. In normal tissues, pericytes have shown to act as “angioregulators” i.e., they stabilize as well as promote angiogenesis; however, their role in tumor microenvironment is yet unclear (Kelly-Goss et al., 2014). Literature cites that they strengthen the blood vessel barrier in co-ordination with endothelial cells or other blood components, thereby preventing vascular leakage. Besides this, they are also known as metastatic stimulators and contribute in accumulation of cancer stem cells within tumor microenvironment (Gerhardt and Betsholtz, 2003; Kang and Shin, 2016; Ferland-McCollough et al., 2017).

Structurally, pericytes are highly elongated, slender, branched cells, with cytoplasmic projections encircling the vessel wall (Diaz-Flores et al., 2009; Sena et al., 2018). They are situated in the basement membrane of tumor blood vessel either as solitary cells or as single-cell layer (Armulik et al., 2011). It is assumed that, in tumor, pericytes are differentiated either from progenitors in the host tissue or from bone-marrow-derived cells (Liu and Ouyang, 2013). In normal angiogenesis, pericytes control the VEGF-mediated endothelial cell proliferation via the direct cell-to-cell contact and paracrine signaling pathways. Through both these mechanisms, pericytes have shown to exert control on proliferation of endothelial cells. Subsequently, they facilitate migration of endothelial cells by degrading the basement membrane and liberate matrix-bound growth factors (Franco et al., 2011; Stapor et al., 2014; Ribeiro and Okamoto, 2015). It has been documented that the endothelial cells on the tip of newly sprouted vascular channel recruit pericytes via secretion of PDGF-BB. This factor activates the pericytes by binding with PDGFR-β receptors expressed on the pericyte surface and induce its migration across the sprouted vascular channel, thereby modulating the pericyte coverage on tumor vessels (Armulik et al., 2011; Minami et al., 2013).

Besides PDGF-BB, other factors such as Angiopoietin-1/Tyrosine Kinase-2 (Ang-1/Tie2), TGF-β, and matrix metalloproteinases (MMP) play a vital role in pericyte-endothelial cell interactions (Chen Z. et al., 2016). Ang-1, reported to be produced by pericyte, binds to Tie2 receptor expressed on endothelium and plays a pivotal role in maturation as well as stabilization of newly formed blood vessels. Similarly, TGF-β, another important factor secreted by endothelial cells for vessel development, facilitate the recruitment of pericytes toward endothelium. Ang-1 primarily induces stabilization of blood vessel by increasing the pericyte coverage over the blood vessels (Fuxe et al., 2011). Similar to Ang-1, Ang-2 is also secreted by endothelial cells activated by cancer-derived modulators, however, it has shown to exert antagonizing effect (Augustin et al., 2009). It is reported that Ang-2 promotes detachment of pericytes from endothelial cells thereby destabilizing the newly formed blood vessels by initiating uncontrolled sprouting of endothelial cells. In case of MMPs, they indirectly promote pericyte recruitment, proliferation and activation by modifying or degrading the basement membrane and enhancing the release of basement membrane bound growth factors (such as VEGF) (Chantrain et al., 2006).

Studies have revealed that pericyte coverage does vary with different malignant tumors (Eberhard et al., 2000). Morikawa et al. (2002) reported that in tumor tissue, pericytes were found to be loosely associated with endothelial cells in both, basement membrane and on tumor micro vessel. Further, their cytoplasmic processes were found to be deeply penetrated into the tumor parenchyma, a distinct feature of tumor vasculature. Yao et al. (2007) demonstrated that in clear cell renal cell carcinoma tumor tissue, pericyte coverage was visibly absent in undifferentiated vessels while for differentiated vessels, loose association, partial or total absence of pericyte coverage was noted. Zhang et al. (2012) have demonstrated that pericyte coverage was found to be more in aggressive cancers such as pancreatic cancer as compared to relatively lesser threatening cancers such as ovarian or colon cancer. Studies by Welén et al. (2008) on prostate cancer cell line LNCaP demonstrated that high pericyte coverage was associated with decrease in tumor metastasis ascribed to reduction in endothelial cell migration. Similarly, Cooke et al. (2012) reported that low pericyte coverage was associated with reduced tumor growth. However, there was increased metastasis in colorectal, prostate, pancreatic and invasive breast cancers. This influential role of pericyte coverage in tumor growth could be due to the overexpression of PDGF-BB by tumors which governs extent of pericyte coverage and inhibit endothelial cell proliferation resulting in reduction of vessel density and overall tumor growth (McCarty et al., 2007; Raza et al., 2010).

Evidently, the researchers have concluded that, both, the aberrant pericyte-endothelial cell interaction and abnormalities in pericyte structure, do contribute to the leakiness of tumor vasculature (Xian et al., 2006). Recent advances in understanding of tumor microenvironment has led to speculation that pericytes may contribute to the acquired resistance to anti-angiogenic therapy (Bergers and Hanahan, 2008). This is believed to be arising either by enhancing endothelial cell survival via crosstalk or by upregulation of the endothelial survival factor Ang-1 or by producing VEGF (Reddy et al., 2008; Welti et al., 2013). Besides this, pericytes owing to the inherent progenitor properties on detachment from tumor vasculature have also shown to differentiate into stromal fibroblasts, a contributor to tumor invasion and metastasis (Hosaka et al., 2016; Paiva et al., 2018).

#### Challenges and opportunities for nano-chemotherapeutics in targeting tumor vasculature

**Figure 3:**
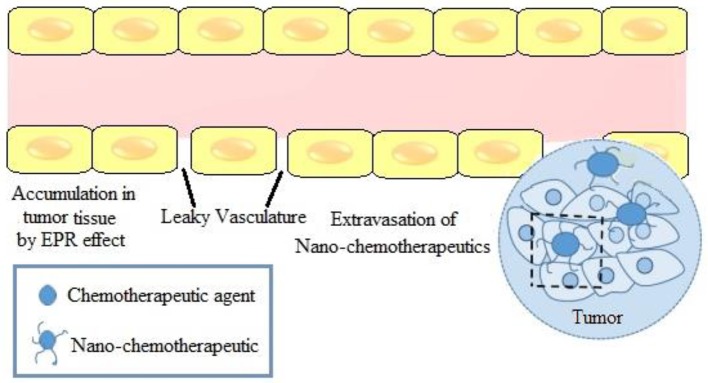
EPR effect in a tumor microenvironment.

For efficient interaction of nano-chemotherapeutics with the cancerous cells in tumor milieu, it is essential that nano-chemotherapeutics are accumulated in the tumor through normal vascular network. Subsequently, their interaction with target cells should be facilitated via selective extravasation from tumor microvasculature and their passage through the ECM (Miao and Huang, 2015). Matsumura and Maeda (1986) were the first to demonstrate the phenomenon of EPR effect of macromolecules in tumor, ascribed to leaky tumor vasculature (Figure 3). Preclinically, ten times higher localization was reported for nanoparticles in the particle size range of 10–100 nm in diameter (Miao and Huang, 2015; Muntimadugu et al., 2017). However, in clinical setting, owing to the complex and heterogenous nature of human tumors, intra-/inter- variability in both tumor characteristics and tumor microenvironment in patient populations, EPR based paradigm approach failed to replicate the success of preclinical studies (Bjrnmalm et al., 2017). Hence, for enhancing the extravasation of nanoparticles, tumor pre-conditioning or priming is being explored. Herein, remodeling of tumor vasculature can be done either by Zhang et al. (2017b) (**i)** Reducing the pericyte coverage, (**ii)** Increasing permeability of tumor microvessels, (**iii)** Tumor vessel dilation or **(iv)** Vascular normalization wherein anti-angiogenic drugs repair the anomalous structure and function of the tumor vasculature network thereby contributing to enhanced tumor perfusion and oxygenation.

The seminal work done by Kano et al. (2007) demonstrated TβR-I (crucial for TGF-β signaling) inhibitor mediated enhanced extravasation of Doxil® (108 nm diameter) as well as micellar adriamycin (65 nm diameter) induced by decreased pericyte coverage of tumor endothelial cells in pre-treated xenografted BxPC3 human pancreatic adenocarcinoma cell line in nude mice. Chaudhuri et al. (2016) showed that at low dose of smoothened inhibitors of hedgehog signaling (erismodegib) pre-treated patient-derived PaCA xenografts promoted extravasation of DOX-loaded sterically-stabilized liposomes (80–100 nm) by promoting formation of immature blood vessels lacking in pericyte coverage with endothelium-poor basement membrane structures. Chauhan et al. (2012) reported that vascular normalization in orthotopic E0771 mammary tumors with anti-VEGF-receptor-2 antibody DC101 showed size-dependent enhancement in tumor reduction, it was superior at lower particle size of ~10 nm (Abraxane) while at higher particle size ~100 nm (Doxil®) it remained unaffected. The reasons ascribed were increase in steric and hydrodynamic barrier with the reduction in pore-size of normalized tumor vessels which benefitted predominantly tumor penetration of smaller particle size. Jiang et al. (2015) suggested controlled dosing of anti-VEGF-receptor-2 antibody DC101 in orthotopically implanted breast adenocarcinoma MCaP0008 cells restored vascular normalization. This ensured enhanced deep tumoral accessibility and penetration of pegylated quantum dots (both 20 and 40 nm) within the tumor stromal matrix. Nevertheless, smaller sized nanoparticles i.e., around 10 nm had superior penetrability across the tumor matrix owing to less diffusional restrictions. Wang et al. (2017) showed that BQ123, a vasodilator which alters an ET-1/ETA transduction pathway as well as blocks the ETA receptor triggered a tumor-specific delivery of photothermal nanomedicine (100 nm) for effective photothermal mediated therapy of tumors.

### Tumor stroma

Broadly, tumor stroma comprises of the following components (Bremnes et al., 2011; Hanahan and Weinberg, 2011; Valkenburg et al., 2018);

**i**. Cellular components such as non-malignant cells widely known as cancer-associated fibroblasts (CAF), host tissue specific specialized mesenchymal stromal cells, osteoblasts, chondrocytes, innate and adaptive immune cells, endothelial cells, and pericytes (Hughes, 2008).
**ii**. Extracellular matrix (ECM) comprises of distinct components having different physical and biochemical properties such as structural proteins (collagen and elastin), specialized proteins (fibrilin, fibronectin, and elastin), proteoglycans, and polysaccharides (hyaluronan) (Özbek et al., 2010). They are further classified into the interstitial matrix and the basement membrane (Xiong and Xu, 2016). The components for interstitial matrix are produced by stromal cells and basement membrane; collectively by epithelial, endothelial, and stromal cells (Lu et al., 2012). Basement membrane is a specialized compact, less porous, thin layers of tumor ECM acting as a supporting scaffold for blood vessels and capillaries. It is situated at the basal surface of epithelial and endothelial cells wherein it plays a crucial role in tissue polarity. It is primarily composed of non fibrillar type IV collagen, laminins, entactins, and proteoglycans (Egeblad et al., 2010; Liotta, 2016).

#### Tumor extracellular matrix

Transformation from normal tissue to tumor tissue is accompanied with profound changes in the tumor microenvironment arising due to enhancement in tumor cell contractility, uncontrolled expansion of the growing tumor tissue, and modifications of the attributes of ECM (Northcott et al., 2018). Generally, tumor ECM (~400 Pa) is found to be stiffer in comparison to normal ECM (150 Pa), and the stiffness is thought to contribute to tumor metastasis, activation of adjoining stromal fibroblast to CAF and can be correlated with the number of tumor associated macrophages (TAM) (Cox and Erler, 2011; Reid et al., 2017). The stiffness of tumor ECM is often associated with relatively high levels of crosslinked collagen (Type I), occurring due to the excessive activity of lysyl oxidase (LOX), as well as increased integrin signaling in the tumor microenvironment mediated by collagen modifying enzymes such as P4HA1, P4HA2, PLOD2, and LOX (Holback and Yeo, 2011). Han et al. (2016) demonstrated that abnormal orientation of collagen I fiber in tumor ECM defined the direction of cell migration and promoted cell breakage into the basement membrane before metastasis. For this, tumor cells via contact guidance, used contractile force to align the ECM fibers perpendicularly to the tumor, thereby induced remodeling of the fibers in the vicinity of tumor (Kraning-Rush and Reinhart-King, 2012). Balcioglu et al. (2016) showed that when 4T1 breast cancer spheroids were kept in contact with collagen based ECM, resulted in re-orientation of surrounding collagen-based ECM network upto five times their radius which further acted as a mechanical cue to guide the movement of microvascular endothelial cells, thereby influencing angiogenesis. Besides this, the increased deposition of ECM in tumor microenvironment governed by various angiogenic growth factors, VEGF, stromal cell derived factor 1, Ang-2, PDGF-B, placental growth factor, and connective tissue growth factors are all associated with solid stress (Danhier et al., 2010; Muntimadugu et al., 2017).

The solid stress constitutes two opposing stress, (**i)** Compressive growth-induced (or residual) stress arising inside the tumor due to both, the expansion of collagen fibers and resistance of proliferating cancer cells as well as activated CAF on compression and (**ii)** Externally applied tensile stress occurring at the interface of tumor-normal tissue exerted by normal host tissue to resist tumor expansion (Kalli and Stylianopoulos, 2018). In ECM, it is reported that owing to its stiffness, cross-linked collagen resists tensile stress, whereas hyaluronan resists compressive stress, attributed to its negatively charged hydrophilic chains which induce electrostatic repulsion and retains water, thereby resulting in a poorly-compressible matrix (Stylianopoulos et al., 2012; Kharaishvili et al., 2014; Pirentis et al., 2015). The stiffness of ECM has shown to promote tumorigenesis by; favoring cell proliferation, triggering epithelial-mesenchymal transition via epithelial cell-ECM interaction, increasing adherence junctions motility, inhibiting apoptosis in a TGF-β1-dependent process and so on. However, there are exceptions such as neuroblastoma cells, colon, prostate cancerous cells, and human tongue squamous carcinoma cells which require low stiffness (Broders-Bondon et al., 2018). Similar observations have been noted for solid stress, Fernández-Sánchez et al. (2015) examined that solid stress via the mechanical activation of β-cat pathway provided mechanical cues in the *in vivo* malignant phenotype of murine colon tissue, for inducing the transformation of surrounding normal epithelial cells into cancer cells. Nguyen et al. (2014) demonstrated that the stiffness due to dense network of collagen-rich fibers also hampered the anticancer activity and conferred resistance against Raf kinase inhibitor, sorafenib.

#### Tumor associated macrophages

TAM are one of the important tumor stromal cells (accounting to ~30% of immune cells) implicated in tumor survival, growth, and metastasis. They are either transformed from the existing macrophages of host tissue or localized to the tumor site from the bone marrow and spleen under the influence of monocyte chemo attractant protein-1 (Binnemars-Postma et al., 2017; Quail and Joyce, 2017). Due to their plastic nature, depending on the type of cytokine exposure, they have been known to exhibit either pro- or anti-inflammatory activities (Quail and Joyce, 2017). Widely investigated TAM include tumor resolving classical M1 macrophage and pro-tumorigenic alternative M2 tumor associated macrophage, together they play pivotal role in tumor vessel abnormalization/normalization in tumor microenvironment (Ngambenjawong et al., 2017). Typically, M1 macrophage normalize tumor vascular network and induce immune response thereby causing the phagocytosis or destruction of tumor cells i.e. tumor regression. In contrast, M2 TAM have anti-inflammatory properties thereby stimulating immunosuppression and formation of abnormal tumor vasculature, indirectly contributing to tumor progression (Jarosz-Biej et al., 2018). It is reported that in an invasive cancer, polarization of M1 to M2 macrophages takes place, making M2 macrophage constitute 50% of the tumor stroma. Functionally, they are known to regulate tumorigenesis, metastasis as well as abnormal angiogenesis, in association with tumor related growth factors, inflammatory components such as cytokines and ECM remodeling molecules such as C-C motif chemokine ligand 2, C-X-C motif chemokine 12, CXCR4, TGF-β, VEGF, PDGF, COX-2, and MMP (Zheng X. et al., 2017). Based on these observations, anti-tumor macrophage therapy is primarily focused either on depletion or re-programming M2 macrophage or regulation of polarization of macrophage (Goswami et al., 2017; Hoves et al., 2018).

#### Cancer-associated fibroblasts

Morphologically and functionally, CAF are a type of mesenchymal cells which are endowed with migratory and contractile properties of myofibroblasts, secreting various factors such as; collagen, cytokines, and chemokines into tumor stroma (Pankova et al., 2016). They play a crucial role in remodeling the tumor stroma by co-ordinating the enzymes responsible for secretion as well as crosslinking of the collagen network of ECM thereby increasing the stiffness of ECM (Clark and Vignjevic, 2015). Further, in presence of tumorigenic hypoxia, they generate collagen reticulation which enhances cancer cell invasiveness, contractility and motility (Pankova et al., 2016). Residing within or across the marginal surface of tumor, the resident fibroblasts on activation via growth factors, direct cell-cell communication, interaction with adhesion molecules, interaction with leukocytes, presence of reactive oxygen species (ROS), and microRNA, transform into CAF (Tao L. et al., 2017). These fibroblasts originate either by alteration of smooth muscle cells or bone marrow-derived stem cells by tumor cells, or transdifferentiation of epithelial cells-to-myofibroblasts, or through endothelial-to-mesenchymal transition (Gascard and Tlsty, 2016). Zhuang et al. (2015) documented CAF induced epithelial-to-mesenchymal transition via growth factor such as TGF-β1. Zhang A. et al. (2017) showed that CAF polarized M2 macrophages mediated through increased production of soluble cytokine macrophage colony-stimulating factor thereby promoting cancer proliferation, invasion, and metastasis in pancreatic ductal adenocarcinoma. Based on the role of CAF in modulating tumor microenvironment, anti-cancer associated fibroblast therapies are being designed to overcome the acquired resistance with anti-cancer therapies.

#### Challenges and opportunities for nano-chemotherapeutics to target tumor stroma

After extravasation, the stiff ECM presents itself as a barrier to the diffusional movement of nano-chemotherapeutics in the interstitial space. The diffusional movement is inversely related to the size of nanoparticles i.e., particles exceeding matrix mesh size ranges 20–40 nm are restricted from diffusing across the ECM in solid tumors. While the nanoparticles nearing the aforementioned mesh size range are hindered to lesser extent and particles less than mesh size range are found to penetrate relatively easily. Besides this, the tortuosity of the interstitial space further lengthens the diffusional path-length of the nanoparticles from normal vessels to tumor cells (Zhang et al., 2017a). It is also believed that the high amount of collagen reduces the hydraulic conductivity thereby decreasing the convective flow in the interstitium (Nichols and Bae, 2014). Hence, tumor matrix degrading enzymes i.e., collagenase and hyaluronidase contribute to improved intratumoral dispersion of nanoparticles by disruption of ECM. This holds true, especially, for collagenase which is found to be more effective in case of nanoparticles, in comparison whereas hyaluronidase is known to facilitate distribution of smaller molecules such as DOX (Au et al., 2016). However, disruption of ECM helps relieve the solid stress by reopening the collapsed tumor vessels but has little or null effect on their leakiness. Similarly, vascular normalization is ineffective for distribution of nano-chemotherapeutics, moreover, it is unable to reduce the solid stress and decompress the tumor vessels compressed by rigid ECM or proliferating cells (Stylianopoulos and Jain, 2013).

Zhang et al. (2017a) demonstrated the use of a selective COX-2 inhibitor, celecoxib in improving the tumor localization of PTX-loaded micelles in a human-derived A549 tumor xenograft nude mice model. Celecoxib was found to modulate tumor microenvironment, by varied mechanisms, viz. reduction of the expression of CAF, distortion of ECM by fibronectin bundle disruption and normalization of tumor vasculature, resulting in improved tumor perfusion. This enabled and improved *in vivo* delivery as well as therapeutic benefits of PTX-loaded micelles. Interestingly, Chen B. et al. (2016) have demonstrated the effect of Tenascin C, a tumor-specific ECM targeted FHKHKSPALSPVGGG peptide-coated liposomal delivery of Navitoclax (NAV), a targeted high-affinity small molecule for priming tumor microenvironment. Tenascin C facilitated tumor localization of liposomal formulation by specifically inducing CAF apoptosis (at a very low dose 5 mg/kg), by reducing interstitial fluid pressure and vascular normalization. The liposomal NAV when used in conjunction or separately with liposomal delivery of DOX was found to improve the tumor localization of DOX. The proposed mechanisms for the enhanced anti-tumor effect included; synergistic anti-cancer activity between DOX and NAV, eradication of CAFs, and deeper penetration of liposomal DOX into tumor tissue. Similarly, Geretti et al. (2015) revealed that the appropriate dose sequencing of cyclophosphamide, enhanced tumoral uptake of HER2-targeted pegylated liposomal DOX in MDA-MB-361 cells, by induction of tumor cell apoptosis, decrease of tumor cell density, reduction of interstitial fluid pressure, with enhanced vascular perfusion.

### Tumor interstitial fluid pressure

In normal tissues, under the influence of capillary hydrostatic pressure there is an extravasation of fluid from the capillaries leading to increased interstitial pressures. However, the pressure is controlled by systemic re-absorption of accumulated interstitial fluid through the post-capillary veins and drainage via lymphatic flow (Scallan et al., 2010). In general, the components of the tumor interstitium can be broadly categorized into the colloid-rich gel domain; comprising predominantly of hydrophilic hyaluronate and proteoglycans, and the colloid-poor fluid-free domain (Omidi and Barar, 2014). Structurally, in cancerous conditions, owing to the irregular geometry, abnormal vasculature, increased ease of transcapillary fluid flow (i.e., vessel leakage), dysfunctional lymphatic drainage system and rapidly proliferating cell burden in the ECM (solid stress), there is constant build-up of interstitial fluid (Lunt et al., 2008; Wiig and Swartz, 2012). The outcome of these solid and fluid pressures in the tumor is the tumor interstitial fluid pressure (Ariffin et al., 2014; Stylianopoulos, 2017). Literature states that for normal tissues interstitial fluid pressure is in the range of −3 to +3 mmHg, whereas for solid malignant tumors, it increases within a range of 5 to 40 mmHg (Baronzio et al., 2012; Simonsen et al., 2012; Yu T. et al., 2013; Wagner and Wiig, 2015). It is experimentally found that the interstitial fluid pressure is greater in the interiors of tumor and precipitously drops across the tumor boundary and surrounding host tissues, possibly in the periphery connected to the normal tissue blood vessels (Siemann and Horsman, 2015). Conversely, the interstitial fluid flow is relatively lower in the tumor interior and is found to be directed toward the tumor boundary and the surrounding tissue (Wu et al., 2013). Increase in interstitial fluid pressure stimulates stretching of tumor cortex, as a consequence, the cell proliferation in the tumor tissue is triggered (Hofmann et al., 2006). Yu T. et al. (2013) demonstrated that high tumor interstitial fluid pressure markedly altered the expression of ~ >1,800 genes associated with invasion and metastasis in SCC-4 and SCC-9 human tongue squamous cell carcinoma cell lines, as a result, there was enhancement in *in vitro* cancer cell proliferation and invasion.

#### Challenges and opportunities for nano-chemotherapeutics to target tumor interstitial fluid pressure

Due to transient high local interstitial fluid pressure, chemotherapeutics are often expelled into systemic circulation from the tumor periphery into adjoining tissues, thereby reducing the efficacy as well as tumor specificity of cancer therapy. Moreover, due to the absence of fluid pressure gradients across the vessel wall and within the tumor, there is a decrease in both, transcapillary flow and convective transport of chemotherapeutics, making diffusion the primary mechanism for transvascular and interstitial transport of drug in tumor (Cairns et al., 2006; Chauhan et al., 2011; Stylianopoulos et al., 2018).

With regards to nano-chemotherapeutics, both diffusion process and outward direction of the interstitial fluid pressure from the core of tumor compromises the distribution as well as localization of nano-chemotherapeutics into tumor tissue (Nakamura et al., 2016). Torosean et al. (2013) demonstrated that uptake of 40 nm fluorescent beads was found to be drastically reduced in tumor having higher interstital pressure arising due to increased collagen content. Hylander et al. (2015) showcased the tumor priming potential of cytotoxic biological therapy Apo2L/TRAIL in lowering interstitial fluid pressure in three different human tumor xenograft models (Colo205, MiaPaca-2 and a patient gastrointestinal adenocarcinoma tumor xenograft). The study revealed that via apoptosis, a single dose of Apo2L/TRAIL drastically lowered the interstitial fluid pressure in treated tumors with broadening of the stromal areas at 48 h. Post treatment, there was significant improvement in tumoral uptake and anti-tumor efficacy of both gemcitabine and liposomal gemcitabine. Interestingly, another study carried out using single dose of tumor priming agent, liposomal imatinib (50 mg/kg) showed reduction in the interstitial pressure in B16 melanoma achieved by inhibition of tumor fibroblasts via blocking PDGF and vascular normalization for almost 50 h. Further, the liposomal tumor priming agent at 20 mg/kg significantly improved the intra-tumoral delivery, accumulation and anti-tumor efficacy of liposomal DOX (Fan et al., 2013).

### Tumor chemical microenvironment

An outcome of aberrant tumor vaculogenesis is inadequate diffusion and perfusion within and across the uncontrollably proliferating tumor tissue leading to generation of two potentially debilitating metabolic crisis i.e., hypoxia and extracellular acidosis (Mistry et al., 2017).

#### Tumor hypoxia

Tumor hypoxia is a consequence of overconsumption of accessible oxygen by rapidly proliferating cells present at the periphery of tumor and also possibly due to inconsistent erythrocyte flux in the abnormal tumor vasculature (Michiels et al., 2016). This variability of oxygen supply arises due to the hindered diffusion of oxygen at a depth beyond 70–150 μm from the peripheral tumor vasculature thereby contributing to an oxygen tension of < 0.1 mmHg (anoxia) to 15 mmHg (Bennewith and Dedhar, 2011; Chitneni et al., 2011; Michiels et al., 2016). Typically, the oxygen concentration in normal tissue and hypoxic solid tumors is found to be in the range of 3–6 and 1–2%, respectively (Ivanovic, 2009; Tian and Bae, 2012). Broadly, tumor hypoxia is classified as chronic hypoxia/diffusion-limited hypoxia and acute cyclic hypoxia/perfusion-limited hypoxia (Dewhirst et al., 1999; Yasui et al., 2010; Patel and Sant, 2016). Chronic hypoxia is a condition characterized by deficit in oxygen level for a prolonged duration (atleast several hours). This is ascribed to longitudinal oxygen gradient i.e., oxygen concentration in the blood remains low, which drastically shortens the length of radial oxygen diffusion in tumor blood vessels causing chronic hypoxia (Matsumoto et al., 2011). In contrast, acute cycling hypoxia affects cancer, endothelial cells and stromal cells adjacent to poorly perfused blood vessels. Herein, the tumor cellular components are exposed to fluctuating periods of re-oxygenation and deep/moderate hypoxia, attributed to cycle of angiogenesis and vascular remodeling activity (occurring over days) and varying erythrocyte flux owing to the redundant tumor vascular network (normally 1–3 fluctuations/h) (Dewhirst, 2007). Pathologically, hypoxia is associated with angiogenesis, metastasis and responsible for acquired resistance to cancer therapy (Muz et al., 2015). In tumor tissues, hypoxia has also shown to induce production of PDGF-BB and other angiogenic factors via HIF-1 (Rankin and Giaccia, 2016). Similarly, hypoxia results in phenotype re-programming of macrophages rendering M1 macrophages unable to present antigens or release pro-angiogenic and immunosuppressive factors, transforming them into anti-inflammatory M2 phenotype (Chanmee et al., 2014).

#### Tumor acidic environment

In normal cells, energy is obtained via oxidative phosphorylation. In contrast, owing to abnormal tumor vasculature and hypoxia, tumor cells derive the energy from the oxygen-independent glycolysis also known as the Warburg effect. The tumor acidic milieu is characterized by increased glucose uptake and fermentation of glucose to lactate. This leads to increase in H^+^ ions production and excretion, however, due to poor tumor vascular perfusion, an acidic extracellular pH (pH_e_) is generated in malignant tumors, pH_e_ of 6.5–6.9 in comparison to physiological pH_e_ of 7.2–7.4. This acidic environment causes efflux of H^+^ ions along the concentration gradient from tumor into adjoining normal non-cancerous tissue, leading to cellular death (Estrella et al., 2013). Besides this, carbonic anhydrase (CA) also contributes to the production of H^+^ ions by catalyzing hydration of excess CO_2_, a by-product of pentose phosphate pathway in tumor (Kato et al., 2013). Thus, a hostile environment encompassing; acidic microenvironment, poor vasculature, proliferating cells, and acidic pH-mediated degradation of ECM by proteinases and subsequent remodeling proves to be detrimental to normal cells. Interestingly, cancerous cells are shown to exhibit better tolerance to the acidic pH by virtue of upregulation of the sodium-hydrogen exchanger or CA (Iessi et al., 2018). Generally, the loss of normal cells and breakdown of ECM has shown to propel the proliferation and invasion of cancerous cells in the vacant space i.e., acidosis contributes to tumor progression via invasion and metastasis (Damaghi et al., 2015). Contrastingly, Warburg phenomenon has also shown to upregulate the expression of crucial transporters for glucose uptake byproducts arising due to glucose metabolism. This enables cancer cells to maintain intracellular pH at 7.4 despite the acidic extracellular pH (Barar and Omidi, 2013; Liberti and Locasale, 2016).

#### Challenges and opportunities for nano-chemotherapeutics to target tumor chemical environment

In general, abnormality of tumor chemical environment is an outcome of aberrant tumor vasculture. Hence, normalizing of tumor vasculature to an extent has shown to counteract tumor hypoxia and tumor acidity, thereby improving the tumoral uptake of nano-chemotherapeutics (Xiao et al., 2017). Herein, the strategies widely explored exploit the tumor microenvironment to deliver environment responsive nano-chemotherapeutics (section Types of Nanocarriers).

## Types of nanocarriers

### Conventional nanocarriers

Taking into consideration the various attributes for facilitating cell-nanoparticle interaction (Fernandes et al., 2018), several nanocarriers have been designed and explored for the treatment as well as management of cancer. Nano-chemotherapeutics provide several advantages over the conventional cancer therapies, such as superior drug efficiency with minimum toxicity, specificity to tumor site, improved drug solubility and stability, greater circulatory half-life of drug, sustained/controlled release, stimuli-activated drug release, nano-chemotherapeutics with imaging modalities and so on. Since the discovery of EPR effect, several formulation scientists have successfully explored nanocarriers which are stable in the blood during their transit to the tumor cells, penetrate deeper into the layers of tumor tissue and accumulate at the tumor site.

**Figure 4:**
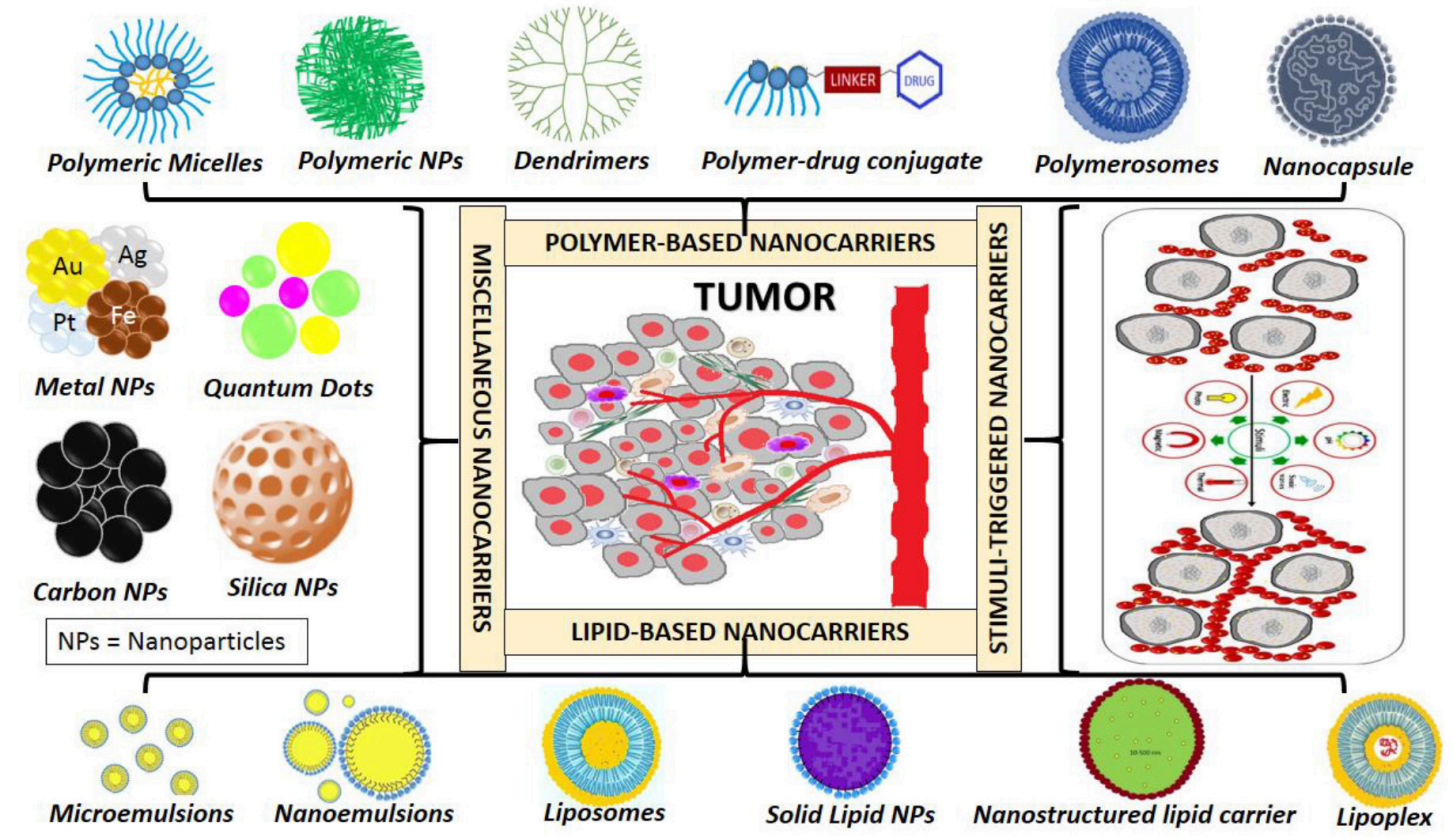
Types of nanocarriers.

Conventionally, nanocarriers may be broadly classified as follows (Figure 4): (a) lipid-based nanosystems viz. liposomes (Zhang X. et al., 2017), solid lipid nanoparticles (Delgado et al., 2012), nanostructured lipid nanocarriers (Shete et al., 2014), nanoemulsions (Izadiyan et al., 2017), lipoplexes (Hattori et al., 2013); phospholipid based microemulsions (Jain et al., 2010) (b) metal-based nanosystems viz. iron oxide, gold, silver, platinum nanoparticles (Bishop et al., 2015); (c) carbon- (Wan et al., 2016) and silica-based (Yu M. et al., 2013) nanosystems; (d) polymer-based nanosystems viz. polymeric nanoparticles (Shen et al., 2013), polymeric micelles (Hao et al., 2015), dendrimers, nanocapsules, polymerosomes, polymer conjugates; (e) surfactant-based nanosystems (Wu et al., 2017); (f) virus-based nanosystems (Ling et al., 2011); (g) biological membrane-based nanosystems and (h) hybrid nanosystems with combination of any above mentioned systems (Shi et al., 2017).

In the last few decades, research has shown that the shape of nano-chemotherapeutics does influence the nanoparticle intravascular, transvascular, and/or interstitial transport as well as their subsequent cellular interaction. This insight about the role of shape of nanocarriers has led to diverse shapes based nano-chemotherapeutics viz., nanospheres, nanoprisms, nanoplates, nanocages, nanorings, nanoboxes, nanostars, nanoflowers, nanodiamonds, nanoshells, nanorods, nanocrystals, nanosheets, nanotubes, nanosnowflakes, and so on (Grazu et al., 2012; Toy et al., 2014). Xie et al. (2017) demonstrated the influence of diverse nanocarrier shapes i.e., nanostar, nanorod, and nanotriangle of methyl-pegylated-anisotropic gold nanoparticles on cellular internalization in RAW264.7 cells (mouse leukaemic monocyte macrophage). The study revealed that the cellular uptake of gold nanoparticles was in the ascending order of nanostar < nanorod < nanotriangle. The dissimilar uptake behavior was ascribed to the endocytotic process. The nanostars were preferrentially internalized via clathrin-mediated endocytotic process. While nanorods were taken up by both via clathrin- and caveolae/lipid raft-mediated endocytosis and were also shown to undergo exocytosis. For nanotriangles, both, clathrin-mediated and cytoskeletal rearrangement enabled dynamin-dependent pathways were found to contribute to superior uptake of nanocarriers.

Pioneering research on nano-chemotherapeutics have reported that the non-spherical nano-chemotherapeutics such as filamentous micelles, nanoneedles, nanorod, or nanodisks displayed greater tumor targeting potential. However spherical nano-chemotherapeutics have shown greater benefits with respect to ease of synthesis, development and evaluation (Truong et al., 2015). The study by Champion and Mitragotri (2006) elucidated the mechanism of uptake of diverse shapes of nano-chemotherapeutics using nonopsonized and IgG-opsonized anisotropic polystyrene particles in continuous alveolar rat macrophage cells NR8383. The researchers designed six different geometric shaped nanocarriers viz., spheres (radius 1.0-12.5 μm), oblate ellipsoids (major axis 4 μm, aspect ratio 4), prolate ellipsoids (major axis 2-6 μm, aspect ratio 1.3–3.0), elliptical disks (major axis 3–14 μm, aspect ratio 2–4, thickness 400–1,000 nm), rectangular disks (major axis 4–8 μm, aspect ratio 1.5–4.5), and UFOs (sphere radius 1.5 μm, ring radius 4 μm). The study documented that for cellular uptake of nano-chemotherapeutics, the angle of contact between the cellular membrane and nanocarriers is the important governing factor. It was stated that the tangent angles of nanocarriers during initial contact either led to orientation-dependent phagocytosis or simple spreading of macrophages without internalization. Study carried by Geng et al. (2007) also demonstrated ten times longer circulation time of PTX-loaded filomicelles of amphiphilic block copolymers of PEG-polyethylethylene or PEG-polycaprolactone than their spherical counterpart in rodents, post intravenous administration. *In vitro* phagocytosis assays were conducted using blood-drawn human neutrophils and human macrophage cell line, THP1. Spherical nanoparticles and short filomicelles (< 4 μm) were taken up by cells more readily than long filomicelles (~18 μm) as the longer filaments were shown to be extended into long chains in the presence of fluid flow. Similarly, Zhou et al. (2013) studied the effect of shape of amphiphilic PEG-*block*-dendritic polylysine-camptothecin conjugates on the cellular uptake, *in vivo* blood clearance, biodistribution and tumor targeting. DOX was used as a tracer to evaluate the cell internalization of nanorod-shaped and nanosphere-shaped conjugates in non-drug resistant cells (MCF-7) and multidrug-resistant cells (MCF-7/ADR). Nanorod-shaped conjugates showed efficient cellular uptake than nanosphere-shaped conjugates owing to their elongated shape. Additionally, long nanorods (>500 nm) exhibited lower blood circulation than medium size nanorods (~1,000 nm) owing to rapid RES clearance and lung accumulation.

### Tumor microenvironment targeted nano-chemotherapeutics

Although, in the last decades, EPR targeting was an underlying paradigm to target nano-chemotherapeutics, there were some limitations involving nano-chemotherapeutics having low molecular weight. In such cases, it was observed that the nano-chemotherapeutics had the tendency to re-enter the systemic circulation via diffusion. Thus, this led to reduced tumor residence time, making it essential to improve the nanotargeting of such chemotherapeutics by considering the overall changing pathophysiological characteristics of the tumoral tissues (Din et al., 2017). Considering this shortcoming, nano-chemotherapeutics targeting to tumor microenvironment has shown to be a promising approach to mitigate drug resistance.

Generally, delivery of effective nano-chemotherapeutics to the tumor microenvironment takes into consideration various endogenous factors such as acidosis, enzyme activity, redox potential, hypoxia, hyperthermia, oxidative stress, high interstitial fluid pressure, and ATP. Additionally, it takes into account, explicit pathophysiological conditions in the tumor microenvironment, such as varying levels of amino acids, functional proteins, DNA fragments and inflammatory cells, such as macrophages, mast cells, lymphocytes, and neutrophils (Chen Y. et al., 2017).

Preclinically, strategies involving pegylated nanocarriers, stimuli-responsive nanocarriers, dual-functional nanocarriers, have demonstrated successful outcomes in abrogating tumor growth by targeting tumor microenvironment. Mostly, these strategies, involve site-specific detachment of PEG linkage (Ding et al., 2015), reversing surface-charge, reduction in particle size, hyperthermia-induced CO_2_ generation (Han et al., 2015), responsiveness to stimuli such as pH (Jiang et al., 2015; Yoshizaki et al., 2016), temperature (Needham et al., 2013; Kokuryo et al., 2017), or responsiveness to external-trigger such as magnetic field (Clares et al., 2013), light (Li et al., 2015), ultrasonic waves, laser (Li et al., 2017), and so on. Broadly, tumor microenvironment targeted nano-chemotherapeutics may be categorized as polymeric-, surfactant-, lipid-, carbon-, silica-, metal-, or metal oxide- based nano-chemotherapeutics.

Targeting the acidic microenvironment of tumor, nano-chemotherapeutics designed using acid-sensitive polymers such as polyethylenimine-Schiff base (Zhao et al., 2017), poly(styrene-co-maleic anhydride) (Dalela et al., 2015), poly(beta-amino ester) (Min et al., 2010), poly-(2-(diisopropylamino)-ethylmethacrylate) (Xu X. et al., 2016), and so on, have been explored. Lee et al. (2008) developed DOX-loaded polymeric micelles comprising of two block copolymers of poly(L-lactic acid)-b-poly(ethylene glycol)-b-poly(L-histidine)-TAT (transactivator of transcription) and poly(L-histidine)-b-poly(ethylene glycol), wherein the micelles were found to protect the TAT and DOX during circulation. Thereafter, in slightly acidic tumor extracellular pH, the micelles were shown to expose TAT to enable the internalization process. Subequently, the internalized copolymers were shown to ionize and disrupt the endosomal membrane facilitating tumor specific DOX release in the xenografted tumors of human ovarian tumor drug-resistant A2780/AD in nude mice. Similarly, Min et al. (2010) designed camptothecin encapsulated pH-responsive micelles comprising of methyl ether PEG-poly(β-amino ester) block copolymer. Within the acidic tumor microenvironment, the micelle disintegrated to release the chemotherapeutic within the MDA-MB231 human breast tumor-bearing mice. Further, the authors explored the tumor microenvironment responsive nature of micelles for noninvasive *in vivo* fluorescence imaging of MDA-MB231 human breast tumor-bearing mice by encapsulating optical imaging fluorescent dye, tetramethylrhodamine isothiocyanate within the micelles. Another approach involved the use of amphiphilic polymer-based self-assembled nanocarriers. These systems have a tendency to undergo protonation-induced hydrophobic-hydrophilic switch in presence of acidic tumor microenvironment. Since such switch destabilizes the assembled nanocarriers, it leads to the release of chemotherapeutics within the tumor microenvironment. Besides this, there are studies which illustrate the role of tumor hypoxia in triggering hydrophobic 2-nitroimidazole-to-hydrophilic 2-aminoimidazole switch leading to destabilization of the nano-chemotherapeutics and subsequent release of chemotherapeutics at the desired site (Chen B. et al., 2017). Zhu et al. (2014) designed tumor reductive environment responsive surface functionalized cationic polylysine endowed with cleavable pegylation and lipophilic histidylation (mPEG-SS-Lysn-r-Hism) for intracellular delivery of siRNA. Cleavable pegylation ensured long circulation of nano-chemotherapeutics in the systemic circulation with selective PEG detachment in response to intracellular tumor reductive microenvironment, facilitating tumoral localization of nano-chemotherapeutics. In this system, histidine conferred lipophilic histidylation for proton sponge effect of imidazole ring and lipophilic benzyl group which led to osmotic swelling of the endosome, disruption of the membrane and promoted release of siRNA intracellularly. In general, PEG-based amphiphilic nano-chemotherapeutics have known to exhibit several advantages such as ability to; overcome multi-drug resistance, co-deliver hydrophobic and hydrophilic drugs, provide longer circulation time of nano-chemotherapeutics, avoid rapid RES clearance and enhance EPR effect (Chen S. et al., 2016). For instance, Mu et al. (2010) developed mixed mPEG-PLA-Pluronic copolymer nano-micelles for better drug bioavailability and to overcome multidrug resistance of docetaxel (DTX).

Modified-liposomes also have been one of the most extensively explored modalities for targeting tumor microenvironment. Liposomes as drug delivery nanocarriers possess advantages of biocompatibility, nonimmunogenicity and delivery of array of chemotherapeutic agents, however they lack tumor specificity which may lead to increased adverse off-target effects. Nevertheless, surface modification of liposomes have enabled multiple functionalities, such as enhanced blood circulation, higher accumulation at tumor site, greater cell internalization and so on (Deshpande et al., 2013). Most prominently, pegylation of liposomes has been done which ensure prolonged systemic circulation of nano-chemotherapeutics. However, there are instances the PEG brush may impede generalized cellular uptake by inhibiting the endosomal escape of liposomes causing degradation of the encapsulated content. Hence, acid-sensitive linkages between PEG chain and hydrophobic moiety such as diortho esters, vinyl esters, cysteine-cleavable lipopolymers, double esters, and hydrazones (stable at pH 7.5, but rapidly hydrolyze at pH < 6) are often incorporated to impart pH-sensitive attribute to liposomes for the specific delivery of cargo in acidic milieu of endocytic vacuole or tumor microenvironment (Deshpande et al., 2013). Liu Y. et al. (2014) have designed DOX- and verapamil-loaded liposomes containing pH-responsive molecule, malachite green carbinol base. This base, on exposure to acidic milieu converted to carbocationic form leading to disorientation of the liposome and target site-specific release of DOX. The anti-cancer activity was further augemented by incorporation of a P-gp inhibitor Verapamil, which aided reversal of multi-drug resistance effect in resistant *in vitro* and *in vivo* breast cancer model. Yan et al. (2015) designed pH-responsive DOX-loaded liposomes coated with glycol chitosan which showed the conversion of anionic nature on the liposomes at physiological pH to cationic nature in acidic extracellular tumor microenvironment. This change in charge facilitated cellular uptake of liposomes in T6-17 tumor-bearing mice thereby enhancing the overall anti-cancer activity. Koren et al. (2012) developed a multifunctional pH-sensitive pegylated long-circulating liposomes of Doxil^®;^, modified with cell-penetrating TAT peptide and cancer-specific mAb 2C5. The immunoliposome contained a pH-sensitive hydrazone bond between long PEG chains and phosphatidylethanolamine component (PE) which degraded specifically at the acidic tumor microenvironment, causing removal of long PEG chains and deshielding the TAT peptide. This multifunctional liposomal system minimized the destruction of non-target cells for much improved anti-cancer therapy.

Apart from pH-sensitivity, hyperthermia-responsive liposomes containing thermosensitive-lipids or polymers have also been studied. Hyperthermia at tumor site has shown to increase tumor permeability and drug uptake by increasing the microvasculature pore size and blood circulation. Prominent advantage of thermosensitive liposomes is its ability to release the contents at phase transition temperature due to melting of liposomes causing open nature and pore-like structures for content release. Park et al. (2014) developed DOX-loaded thermosensitive liposomes of 1,2-dipalmitoyl-sn-glycero-3-phosphocholine, 1,2-distearoyl-sn-glycero-3-phosphoethanolamine-N-[methoxy(polyethyleneglycol)-2000], cholesterol, and a fatty acid conjugated elastin-like polypeptide in the molar ratio, 55:2:15:0.41, respectively. The research protocol contained induction of local heating (42°C) of 30 min prior to i.v. injection, at the tumor site of tumor-bearing BALB/c nude mice for enhanced EPR effect. The study outcomes suggested better accumulation of DOX-loaded liposomes when compared to free DOX-treated group, both, in presence and absence of preheating. DOX-loaded liposomes under preheated conditions exhibited 5-fold, 11-fold, and 31-fold increase in drug accumulation at 6 h after i.v. injection when compared to DOX-loaded liposomes without preheating, free DOX-treated group without preheating, and free-DOX treated group with preheating, respectively. Similarly, Achim et al. (2009) developed DOX-loaded liposomes composed of thermosensitive lipids; dipalmitoylphosphatidylcholine (DPPC) and distearoylphosphatidylcholine (DSPC) alongwith cholesterol in the molar ratio, 26:4:6, respectively; DOX-loaded lyso-thermosensitive liposomes composed of 1-palmitoyl-2-hydroxy-3-glicerophosphatidylcholine, DPPC, DSPC and cholesterol in the molar ratio of 2:24:4:6, respectively and DOX-loaded non-thermosensitive liposomes composed of L-α-phosphatidylcholine and cholesterol in the molar ratio of 30:6, respectively. Though, DPPC showed gel-to-liquid phase transition temperature (Tc) at a clinically attainable local hyperthermia, 41.5°C, it was associated with poor rate and reduced amount of drug release. Hence, it is used in combination with other lipids such as DSPC having higher Tc at 54.9°C.

Mesoporous silica nanochemotherapeutics as drug delivery carriers have also been explored for its ability to have higher drug loading (about 60%). However, they have limitations, due to the porous structure of the silica nanoparticles there is often leakage of drugs and undesirable burst release. To address this issue, tumor microenvironment-responsive mesoporous silica nano-chemotherapeutics have been designed, wherein stimuli-triggered caps may be provided for protecting the drug-loaded pores in the mesoporous silica nano-chemotherapeutics and promoting drug release only in the tumor microenvironment (Schlossbauer et al., 2009; Liu et al., 2015). Various molecules which behave as gatekeepers include ferric oxide, avidin, peptides, human serum albumin, β-cyclodextrin, gelatin etc., may be incorporated during the designing of silica-based nano-chemotherapeutics. Removal of these gatekeepers, triggered by tumor microenvironment-related factors such as acidic pH, redox potential, over-expressed enzymes have shown advantages over non-triggered systems (Chen B. et al., 2017).

Metal or metal oxide nano-chemotherapeutics have also shown potential in the tumor microenvironment. For instance, Crayton and Tsourkas (2011) explored the pH-titratable superparamagnetic iron oxide for accumulation of nanoparticles in the tumor microenvironment. The metal oxide nanoparticles were conjugated with glycol chitosan, a water-soluble polymer with pH-titratable charge, demonstrating *in vitro* pH-dependent cellular association. Zhong et al. (2013) developed hybrid micelles using PEG-PLA (organic-inorganic) copolymers, which were further coated with gold nanorod in order to improve the stability of nanocarriers during systemic circulation. In addition to this, these systems were designed to release the anti-tumor agent on exposure to NIR radiation due to the phase transition of PLA induced by the photothermal effect.

Few examples of tumor microenvironment governed conventional nano-chemotherapeutics are shown in Table 1.

**Table 1:** Few examples of tumor microenvironment governed conventional nano-chemotherapeutics targeted for tumor therapy.

**Tumor microenvironment**	**Type of Nano carriers**	**Reference(s)**
Acidic pH	Iron oxide nanoparticles	Crayton and Tsourkas, 2011
	Liposomes	Yan et al., 2015
	Silica nanoparticles	Deng et al., 2011
	Polymeric nanoparticles	Dalela et al., 2015; Sun et al., 2015; Hung et al., 2016; Xu C. F. et al., 2016
	Polymeric micelles	Hu et al., 2013; Ouahab et al., 2014
Hypoxia	Polymeric nanoparticles	Perche et al., 2014; Thambi et al., 2014
Oxidative stress	Polymeric nanoparticles	Kwon et al., 2013
Hyperthermia	Quantum dots	Tao W. et al., 2017
	Iron oxide nanocubes	Guardia et al., 2012
	Liposomes	Peng et al., 2014
	Magnetic nanoparticles	Yu et al., 2014
ATP	Micelles	Naito et al., 2012
	Polymeric Nanogel	Mo et al., 2014
	Microcapsules	Liao et al., 2015
PEG detachment	Polymeric nanocarriers	Dong et al., 2015
	Liposomes	Yan et al., 2015
	Silica nanoparticles	He et al., 2016
	Polymeric nanoparticles	Dreaden et al., 2014
	Layer-by-layer films on nanoparticles	Deng et al., 2013
Particle-size shrinkage	Nanoparticles	Ruan et al., 2015
	Polymeric nanoparticles with Dendrimers	Li et al., 2016
	Nanogel	Ju et al., 2014; Zan et al., 2014
	Gold nanoparticles	Huang et al., 2012; Huo et al., 2013
	Lipid-dendrimer nanoassembly	Sun et al., 2014

Recently, multistage nano-chemotherapeutic approach has evolved which has helped in reducing the shortcomings of conventional nanotherapy such as drug resistance, circulatory half-life of the actives, heterogeneity of cancer cells, and suppression of immunity by the tumor microenvironment (Conniot et al., 2014). As mentioned earlier, tumor microenvironment over-expresses various receptors, possesses higher redox potential and increases levels of certain enzymes. Thus, the approaches further discussed in this review are focused on ligand-mediated, redox-responsive, and enzyme-mediated nano-chemotherapeutics.

#### Ligand-mediated nano-chemotherapeutics

**Figure 5:**
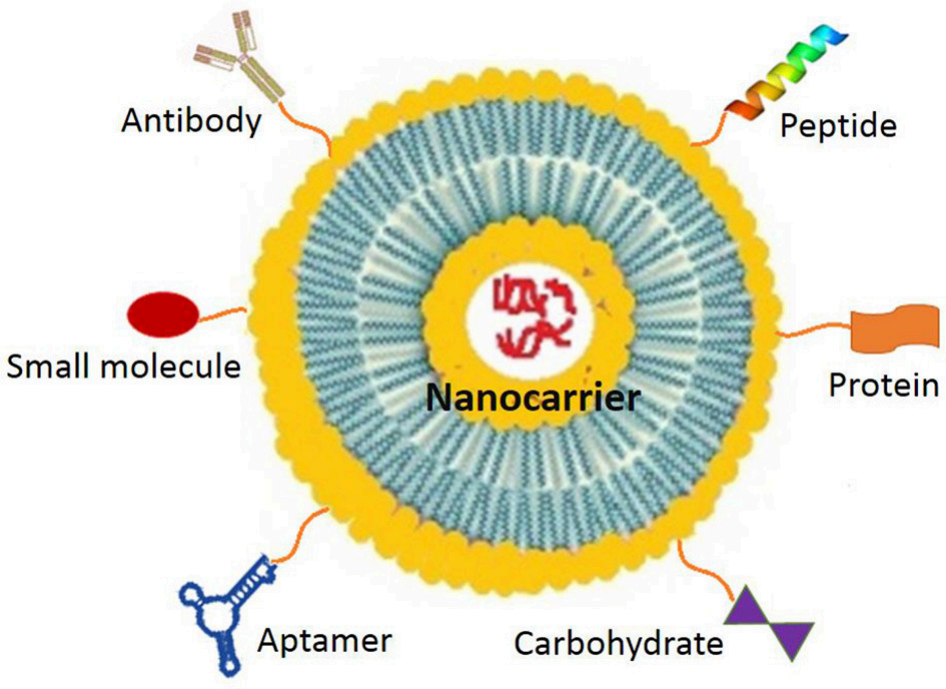
Different types of ligands for targeting nano-chemotherapeutics.

Ligand-mediated nano-chemotherapeutics have shown promising outcomes due to their passive as well as predominant active targeting. However, they lack clinical outcome due to diverse expression of membrane receptors between patients, type- and stage- of tumor and prevention of penetration of extravasated nano-chemotherapeutics due to peripheral receptor-ligand interactions with the tumor cells (Chen B. et al., 2017). For successful active targeting, it is desirable that the ligand functionalized on the surface of the nano-chemotherapeutics have selectivity toward the receptors over-expressed homogenously and specifically by tumor cells at all tumor sites. In addition to this, the selected ligand should either bind or modify the tumor core vasculature (Lammers et al., 2008) or facilitate cellular internalization of nano-chemotherapeutics (Cho et al., 2008). For effective internalization of ligand-mediated nano-chemotherapeutics (Figure 5), various over-expressed internalization-prone receptors on tumor cell surface have been explored, which include, transferrin receptor, folate receptor, epidermal growth factor receptor (EGFR) and fibroblast growth factor receptor (FGFR). Besides this, internalization-prone receptors are also over-expressed in the cytoplasm and nucleus of the tumor cell namely, peroxisome proliferator-activated receptor γ (PPAR-γ). Similarly, in tumor microenvironments or tumor vasculature or endothelium of tumor neovasculature, overexpressed receptors include; VEGF receptor, α_ν_β_3_ integrin receptor, vascular cell adhesion molecule-1 (VCAM-1) glycoprotein and MMPs (Danhier et al., 2010).

Literature cites that targeting ligands could also include monoclonal antibodies or antibodies, such as trastuzumab, bevacizumab, etaracizumab, Ibritumomab tiuxetan, denosumab, or antibody fragments such as fragment antigen-binding (Fab') or single-chain fragment variable (scFv). Surface functionalization using antibody and their fragments have paved way for immunotherapy in oncology. It is proposed that during functionalization use of fragments instead of whole antibody is more advisable to preclude the risk of antibody inactivation. Further, reduced size of the ligand has shown to minimize untoward immune response and promote effective targeted delivery. Besides this, non-antibody ligands, peptides such as endostatin, aptamers, small molecules such as folates, carbohydrates, affibody, and so on have also been explored. Widely known techniques of functionalization of nano-chemotherapeutics employ chemical interaction through covalent or non-covalent bonding between nanocarrier and functional group. For instance, in case of liposomes, functionalization may involve formation of amide bonds; disulfide bonds; thioether bonds or PEG-linkages (Riaz et al., 2018).

Using this strategy, endothelial cell receptor (such as integrin α_v_β_3_) targeted ligand-nano-chemotherapeutics has been developed having the ability to perturb angiogenic vessels and tumoric cells. This approach has shown to enable bypass of biological tumor barriers, mitigation of drug resistance due to endothelial cells genetic stability, and generation of verstaile nano-chemotherapeutics having widespread application in several tumor types (Ji et al., 2013). Kibria et al. (2013) investigated the effect of size of arginine-glycine-aspartic acid peptide (RGD) pegylated liposome on the target specific delivery of DOX to integrin α_v_β_3_-expressing tumor endothelial cells. The study was carried out for two size populations; average diameter ~100 nm (small size) and ~300 nm (large size) and marketed product Doxil® (~100 nm in diameter) in DOX resistant OSRC-2 (Renal cell carcinoma) tumor xenografts. The study results verified that the size of liposomes strongly influenced ligand-receptor interaction, internalization of liposomes into the cell and distribution in the tumor microenvironment. Despite the accumulation of Doxil® in the tumor cells, the marketed formulation failed to suppress tumor growth. Similarly, owing to extravasation of small-sized ligand-mediated and pegylated liposomes to tumor cells, there was lack of appreciable anti-tumor activity. Interestingly, large-sized ligand mediated and pegylated liposomes having minimized EPR effect demonstrated superior targeting to tumor endothelial cells via integrin α_v_β_3_. Consequently, it exhibited anti-angiogenic activity on tumor vasculature and thereby reduced tumor growth. Babu et al. (2017) fabricated RGD-modified polylactic acid-co-glycolic acid-chitosan nanoparticles for integrin α_v_β_3_ receptor targeted PTX delivery in lung cancer cells. The approach resulted in superior uptake via integrin receptor-mediated endocytosis, cell-specific delivery, elicited improved apoptosis, and induced G2/M cell cycle arrest.

Another approach involves inhibition of VEGF or VEGFR binding to regulate tumor angiogenesis and neovascularization (Ji et al., 2013). Feng et al. (2014) developed VEGF-targeted siRNA- (siVEGF) and PTX-loaded vapreotide-modified core-shell type nanoparticles for improved intracellular siRNA accumulation and VEGF down-regulation in human breast cancer MCF-7 cells as compared to non-ligand nano-chemotherapeutics. *In vivo* study results of ligand-based nano-chemotherapeutics demonstrated enhanced tumor tissue localization and efficient inhibition of tumor growth via siVEGF silencing induced neovascularization inhibition. In another study, siRNA- and DTX-loaded two receptor-specific peptides-modified liposomes [i.e., LDL receptor-related protein receptor (Angiopep-2) and neuropilin-1 receptor (tLyP-1)] were found to be having superior brain tumor targeting and tumor penetration (Yang et al., 2014). Shein et al. (2016) focused on studying the effect of VEGF- and VEGFR2-targeted liposomes for delivery of cisplatin to C6 and U-87 MG glioma cells. The drug and antibody-conjugated liposomes exhibited prolonged release *in vivo*, superior affinity to antigens and enhanced uptake by the glioma cell lines. Li et al. (2015) developed a human epidermal growth factor receptor-2 antibody-conjugated drug delivery system, using near-infrared (NIR) light-sensitive DOX-loaded liposomes and hollow gold nanospheres. These systems showed superior and specific binding and selective toxicity to human epidermal growth factor receptor-2-positive tumor cells. Combination of liposomes with NIR laser irradiation led to 92.7% tumor inhibition due to enhanced accumulation in tumors, which was further attributed to photo-thermal-chemotherapy. Furthermore, vascular cell adhesion molecule-1 (VCAM-1), an angiogenic factor and MT1-MMP, an enzyme, both expressed on the tumor endothelial cells in several tumor types are also considered as potential broad-spectrum targets for ligand-mediated nano-chemotherapeutics.

Hypoxia, a tumor condition characterized by overexpressing CA (e.g., their transmembrane isoforms which include CA IX and CA XII) in the tumor microenvironment (Mahon et al., 2015). Thus, the widely known approach to counteract this condition involves inhibition of CA activity. Stiti et al. (2008) developed CA inhibitor coated gold nanoparticles for combating hypoxic tumors by selectively inhibiting tumor-associated CA isoform IX over cytosolic isozymes I and II. For *in vivo* inhibition of transmembrane vs. cystolic isozymes, the researchers investigated the penetrability of sulphonamide inhibitors and CA inhibitors using red blood cells (RBC) as experimental model. Study results revealed higher diffusibility of sulfonamide inhibitors through the RBC membranes, while CA inhibitor coated gold nanoparticles showed membrane impermeability. This impermeable nature was found to be contributing factor for selective inhibition of CA IX expressing tumors. Similarly, Krall et al. (2014) developed a small molecule-disulfide-linked-drug conjugate for inhibiting CA IX expressed in renal cell carcinoma SKRC52. Herein, maytansinoid DM1 a chemotherapeutic conjugated to CA IX specific ligand, a derivative of acetazolamide showed enhanced internalization of chemotherapeutic agent and exhibited a potent antitumor effect. Lin et al. (2017) also surface functionalized triptolide-loaded liposomes with anti-CA IX antibody to target CA IX-positive human non-small cell lung cancer cells (A549) and A549 tumor spheroids, resulting in the efficient cell apoptosis as compared to free drug and non-targeted liposomes. In a study by Takacova et al. (2016), instead of surface functionalization, the scientists encapsulated the monoclonal antibody M75 into alginate microbeads and sodium alginate-cellulose sulfate-poly(methylene-co-guanidine) based microcapsules to knock down the CA IX expressed by tumor cells.

Cell-penetrating peptides (CPPs) mediated drug delivery has emerged as a promising strategy to deliver nano-chemotherapeutics through tissue and cell membranes either using an energy-dependent or -independent process. Generally, they are conjugated to nano-chemotherapeutics either via stable, non-covalent complexes or via covalent bond. However, they have limitations, some peptides lack stability as they are scavenged (especially, cationic peptides) by reticulo-endothelial system (RES) and also specificity deficient for cancerous cell targeting, leading to toxicity. To circumvent this issue, new generation multistage ligand-mediated nano-chemotherapeutics that are responsive to tumor microenvironment are designed, which are classified as follows: (a) activatable CPPs (ACPPs), (b) de-shielding systems, (c) “pop-up” systems, and (d) trojan-horse targeting systems (for detailed review, refer, Chen B. et al., 2017; Kebebe et al., 2018). In ACPPs, tumor microenvironment responsive materials are exploited to display CPPs either at lower pH or in presence of overexpression of ECM development-remodeling proteases, or in response to external stimuli of heat or light to a disease site (MacEwan and Chilkoti, 2013). Lee et al. (2017) synthesized poly-l-lysine-based pH-controllable CPPs which undergo pH-dependent conformational transition and display CPPs at the target site. It was reported that at physiological pH, the electrostatic attractions existing between carboxylate and protonated amine groups in side chain of CPPs contributed to its low helical tendency, thereby preventing unselective cellular internalization. However, in tumor acidic microenvironment, the helical conformation was attained enabling cell-penetrating properties at specific cancer sites (Lee et al., 2017). Zhang Y. et al. (2017) developed DOX- and vincristine-loaded liposomes, which were modified with 2 ligands, i.e., T7, seven-peptide ligand of transferrin receptors (TfR) and ^D^A7R, d-peptide ligand of VEGFR 2 for efficient targeting to glioma. The dual peptide conjugated liposomes were capable of circumventing blood brain barrier and possess glioma-homing property due to the presence of the dual peptides. Singh et al. (2016) designed RGD-conjugated d-alpha-tocopheryl PEG 1000 succinate (TPGS) theranostic liposomes comprising of DTX and quantum dots, showing potential for brain tumor treatment and imaging. On similar lines, Chen Y. et al. (2017) developed PTX-loaded mixed micelles of vitamin E succinate-grafted-chitosan oligosaccharide/RGD-conjugated TPGS for human glioma U87MG tumor targeting. In addition to the earlier conventional nano-chemotherapeutics developed by Zhong et al. (2013), they developed multistage nano-chemotherapeutics by functionalizing the gold nanorod coated micelles with a cRGD ligand. This advancement overcome MDR effect by improving the tumor penetration of the active moiety (Zhong et al., 2014). Zhao et al. (2016) also developed tumor-specific DOX-loaded pH-responsive liposomes with peptide H7K(R2)2 as a targeting ligand.

Combining the benefits of ligand-mediated nano-chemotherapeutics with stimuli responsiveness, Zang et al. (2016) successfully developed a pH-sensitive cholesterol-Schiff base-PEG-modified cationic liposome-siRNA complex conjugated with recombinant humanized anti-EphA10 antibody. The study results revealed that the complex was able to escape from the endo-lysosomal organelle and release the active drug into cytoplasm, which was evident by *in vivo* studies, wherein the complex was able to target the tumor site. Similarly, Al-Ahmady et al. (2014) designed an approach combining the benefits of ligand-mediated response with tumor induced-hyperthermia. In the study, DOX-loaded thermo-sensitive liposomes were designed and conjugated with hCTMO1 monoclonal antibody (anti-MUC-1). Receptor-mediated cellular uptake and cytotoxic effects of antibody-targeted thermo-sensitive liposomes was observed in MUC-1 over-expressed breast cancer cells (MDA-MB-435), post-heating for 1 h at 42°C.

Other agents such as hyaluronic acid (HA), transferrin and aptamers have also been reported as ligands to confer nano-chemotherapeutics with tumor targeting ability. Zheng T. et al. (2017) developed negatively charged HA-coated silica nanoparticles loaded with two chemotherapeutic agents, candesartan, and trastuzumab. The ligand-mediated delivery system facilitated cell internalization of nano-chemotherapeutics within tumor cells of an ovarian cancer. In a study carried out by Han et al. (2014), they successfully developed surface-modified, co-encapsulated solid lipid nanoparticles containing green fluorescence protein plasmid and DOX for multifunctional delivery to lung cancer cells (human alveolar adenocarcinoma cell line-A549 cells). Transferrin-containing ligands were surface-coated on the vectors and the study results demonstrated superior lung cancer gene therapy. Qin et al. (2014) designed dual cyclic RGD peptide- and transferrin-conjugated PTX-loaded liposomes for targeting the blood-brain-barrier and targeting to brain glioma cell. Wang et al. (2015) successfully encapsulated MicroRNA-34a into a S6 aptamer-conjugated dendrimer to create lung cancer-targeted gene delivery nanoparticles. Aptamer-conjugated nano-chemotherapeutics improved cellular uptake and gene transfection efficiency of the dendrimeric nano-chemotherapeutics in cultured non-small cell lung cancer cells. The nano-chemotherapeutics also inhibited cell growth, migration, and invasion and induced apoptosis when compared to non-targeted nanoparticles, thus proving better systems for tumor targeting.

#### Redox-responsive nano-chemotherapeutics

**Figure 6:**
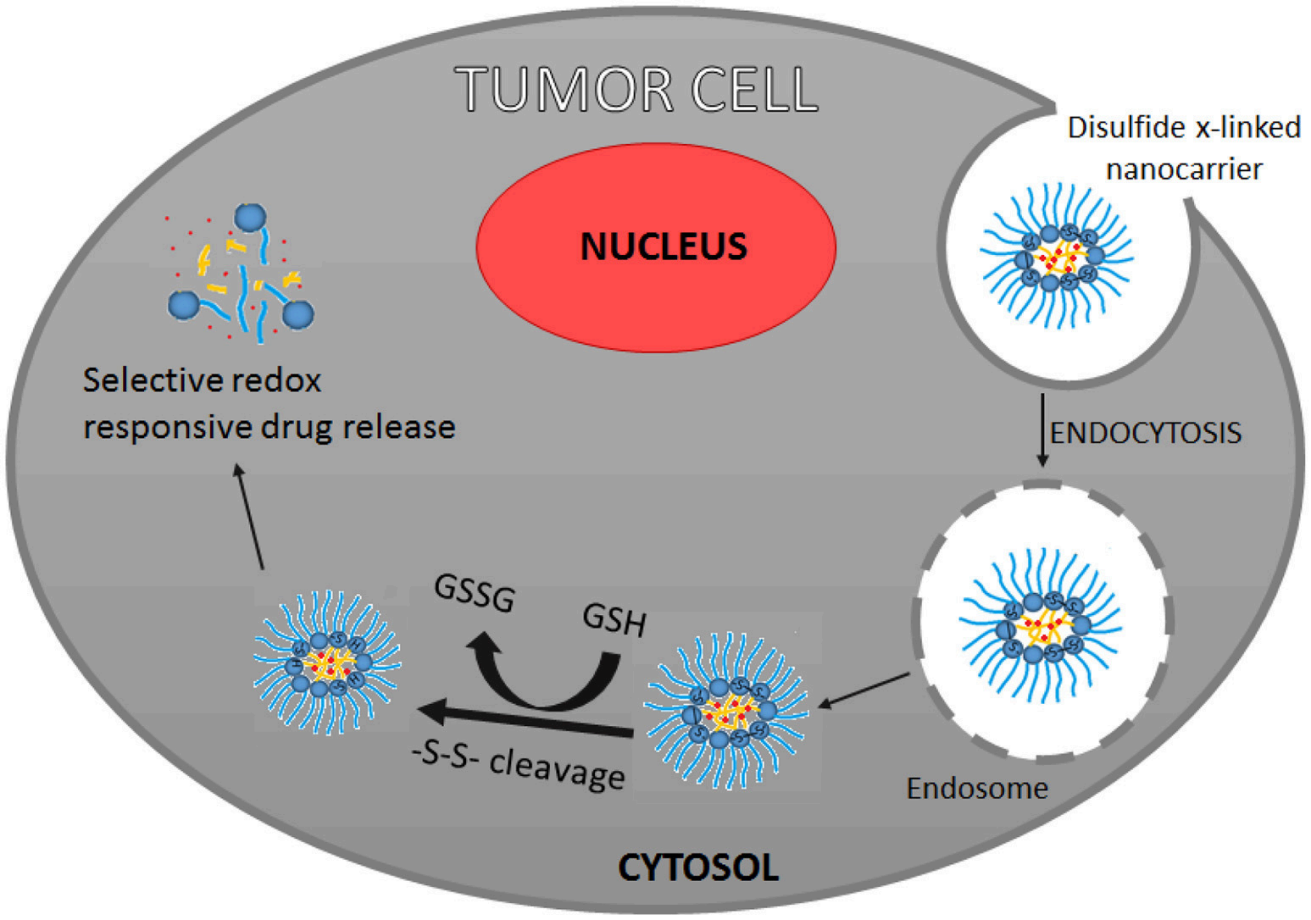
Redox responsive nanocarriers.

Redox potential, an intracellular stimulus, is the outcome of reduced glutathione concentration in extracellular space as compared to intracellular space, whose amplification causes cleavage of disulfide bond by glutathione leading to disassembly and degradation of nano-chemotherapeutics, followed by release of the chemotherapeutics (Figure 6) (Chen B. et al., 2017). Several researchers have designed nano-chemotherapeutics having disulphide bonds rendering them redox-responsive within tumor microenvironment. Sun et al. (2010) developed amphiphilic polyamide amine-g-PEG graft copolymer containing disulfide linkages which degraded in presence of reducing agent, dithiothreitol, leading to disassembly of micelles and complete release of chemotherapeutics within 10 h as compared to 25% release in 24 h. Wu L. et al. (2016) developed DOX-loaded zwitterionic nanoparticles comprising of dextran using succinic acid and crosslinked with cystamine which showed rapid internalization of Hela cells, making it suitable alternative to target tumor reductive environment. Similarly, Hou et al. (2016) fabricated HA-modified single-walled carbon nanotubes wherein the HA was further conjugated to DOX by disulphide bonds making the nano-chemotherapeutics sensitive to redox potential. Whereas, Lin et al. (2016) developed bioreducible DOX-loaded chitosan-based nano-chemotherapeutics with HA for production of intracellular glutathione-triggered reactive oxygen species and simultaneous release of chemotherapeutics. Shi et al. (2014) developed DOX-loaded star-shaped micellar system made up of amphiphilic co-polymer crosslinked with phenylboronic acid-functionalized reduction-sensitive amphiphilic co-polymer having tumor targeting ability. Yin et al. (2018) designed redox-responsive, estrogen-functionalized liposomes covalently tethered to biocompatible chotooligosaccharides, via disulphide links, for intracellular delivery to osteosarcoma MG63 cells. The liposomes demonstrated high drug loading with average diameter of ~110 nm which were capable of exploiting the EPR effect. These multifunctional liposomes showed immediate cellular uptake via estrogen-receptors followed by rapid drug release due to the reduction of disulphide bond by intracellular glutathione concentration. Maggini et al. (2016) explored the redox-mediated drug release by designing redox-responsive mesoporous organo-silica nanoparticles containing disulphide bridges inside glioma C6 cancer cells. Similarly, Wu M. et al. (2016) developed redox-sensitive huge pore-sized hollow mesoporous organosilica nanoparticles for concurrent delivery of P-gp modulator siRNA and chemotherapeutics for reversal of MDR effect of tumor cells. Koo et al. (2012) fabricated a multi-unit nano-chemotherapeutics i.e., core-shell-corona micelle comprising of redox-responsive shell-specific cross-links. The DTX-loaded core shell composed of poly(L-phenylalanine), while the mid-shell was made up of poly(L-lysine), providing the disulphide bonds for reductive cleavage by the intracellular glutathione and finally the PEG-based outer corona for enhanced systemic circulation of the nano-chemotherapeutics. Exemplifying the potentials of multi-unit nano-chemotherapeutics, Li et al. (2016) fabricated a pH-sensitive cluster nanoparticles combined with platinum prodrug-conjugated poly(amidoamine) dendrimers possessing multiple-trigger induced drug release in the tumor microenvironment. Herein, the various tumoral barriers were disrupted sequentially; the strategy involved tumor localization by promoting size-dependent extravastion of ~100 nm polymer-based cluster nanoparticles. Following which, the nanocarriers shrunk in size to ~5 nm platinum-linked dendrimer in response to acidic tumor microenvironment. This facilitated tumor penetration and cell internalization followed by rapid reduction of dendrimer-prodrug by the intracellular glutathione in the cytosol to release the chemotherapeutic agent. Similarly, Chi et al. (2017) designed PEG stabilized drug-loaded liposomes conjugated with cholesterol using reducible disulfide linkages wherein the cationic liposomes were coated with a CD44 sensitive ligand i.e., HA. The redox-sensitive liposomes showed 60% release of actives in presence of glutathione, when compared to the redox-insensitive liposomes. This multi-triggered concept provided CD44-mediated tumoral delivery with improved systemic circulation and cancer cell specific glutathione-triggered cytoplasmic release of chemotherapeutics.

Yang et al. (2016) also developed a dual-stimulus system, exploiting two-triggers, i.e. hyperthermia and reductive tumor microenvironment. The researchers fabricated thermosensitive liposome conjugated with Asparagine-Glycine-Arginine (NGR) peptide as ligand and reducible siRNA-CPPs for tumor-specific siRNA transfection in order to prevent CPP degradation and reduction of disulphide linkages, *in vivo*. In comparison to conventional siRNA-CPPs, the dual-stimulus nano-chemotherapeutics under hyperthermic condition showed 2-fold gene silencing efficiency and 3-fold higher antitumor activity in HT-1080 xenograft murine model. This dual responsive nature is well understood by a study carried out by Ghosh et al. (2009), wherein the researchers developed a supramolecular complex of cationic surfactant and anionic disulfide containing polyelectrolyte formed micelles having redox- and acidic pH-responsive attributes. The study ascribed the responsive nature to the micellar changes arising due to reductive disulfide bond cleavage-mediated conversion of polyvalent-to-monovalent interaction and acidic pH-mediated reduced electrostatic interactions between polymer and surfactant.

#### Enzyme-mediated nano-chemotherapeutics

Over-expression of enzymes such as peptidases (e.g., aminopeptidase), proteases (e.g., MMP and cathepsin B), and lipases (e.g., phospholipase A2) by the stromal cells or tumor cells for their growth, angiogenesis, invasion and metastasis have paved way for developing enzyme-mediated nano-chemotherapeutics (Gullotti and Yeo, 2009; Chen B. et al., 2017). Roy et al. (2010) reviewed the use of different enzyme-responsive polymers in designing the nano-chemotherapeutics which are susceptible to enzymes overexpressed by tumor cells. Yamada et al. (2010) used the protease activity of MMP2 to selectively deliver PTX to tumor site. Two different prodrugs of PTX (i.e., PTX-2'AcG and PTX-7AcG wherein the 2'- and 7-hydroxyl groups were conjugated to C-terminus of glutamine of octapeptide) were found to be vulnerable to cleavage by the MMP2 at tumor site, to release PTX. In addition to this, the presence of octapeptide minimized the affinity of the drug to MDR1, thus abrogating MDR effect. Hatakeyama et al. (2007) developed an enzymatically cleavable PEG-peptide-lipid ternary conjugate based carrier of a gene for cancer therapy. The *in vitro* study showed that the MMP-sensitive liposomes exhibited superior gene transfection efficiency in overexpressed MMPs in comparison to MMP-insensitive liposomes. Similarly, Chau et al. (2006a,b), designed a dextran-peptide-methotrexate conjugate for tumor site specific release of chemotherapeutics in response to tumor-associated enzymes i.e., MMP-2 and MMP-9 via cleavage of peptide linkage. However, their study revealed a reduction in tumor growth by nano-chemotherapeutics containing MMP-sensitive and MMP-insensitive linkers as compared to chemotherapeutic alone. The probable reason cited for this anomalous behavior was the endocytic uptake of the conjugate prior to cleavage by the enzymes. Harnoy et al. (2014) synthesized enzyme-responsive amphiphilic hybrids containing linear PEG and a stimuli-responsive dendron with enzyme-cleavable hydrophobic end groups, with a potential to self-assemble in water as smart micellar nano-chemotherapeutics for encapsulation of hydrophobic chemotherapeutics. The hybrids accommodated phenyl acetamide as hydrophobic end groups cleavable by the enzyme penicilin G amidase, which on enzyme-induced degradation, resulted in the destabilization of micelles and formation of monomeric hydrophilic hybrids, with subsequesnt release of chemotherapeutics.

Another protease enzyme that is over-expressed in the extracellular tumor microenvironment by stromal, TAM and endothelial cells in tumors is Legumain, making it potential stimulus in targeting the tumor stroma. Ruan et al. (2016) successfully developed enzyme-responsive nanoplatforms that activated the accumulation of gold nanoparticles using legumain for improved localization of chemotherapeutics in brain tumors. Researchers designed a mixed surface decorated gold nanoparticles (i.e., AuNPs-A&C) comprised of Ala-Ala-Asn-Cys-Lys modified nanoparticles (termed as AuNPs-AK) and 2-cyano-6-aminobenzothiazole modified nanoparticles (termed as AuNPs-CABT). In presence of enzyme legumain, AuNPs-AK hydrolysed to expose 1,2-thiolamino groups, which further underwent click cycloaddition with contiguous cyano group on AuNPs-CABT, forming gold nanoparticle aggregates. These systems possessed enhanced retention capability in the tumor cell due to the inhibition of nanoparticle exocytosis and prevention of backflow into the systemic circulation. Using the similar concept, Xiao et al. (2017) developed an enzyme-induced size-changeable gold nanoparticles loaded with cediranib, a tumor vessel normalizing agent for breast cancer tumor imaging and therapy. Ruan et al. (2017) further explored dual-functional gold nanoparticles with enzyme-responsiveness and integrin α_v_β_3_-ligand-mediated targeting. Liu Z. et al. (2014) fabricated a legumain protease-activated TAT-liposome, wherein the substrate for endoprotease legumain, alanine-alanineasparagine, was linked to the 4th lysine of TAT. The substrate was susceptible to breakage by legumain enzyme overexpressed in tumor microenvironment, leading to tumor targeting of liposomes to the desired site.

Hyaluronidase is another enzyme expressed 20–1000 times higher by the tumor cells having capability of degrading HA-coated nano-chemotherapeutics (Li and Xie, 2017). Yim et al. (2013) designed a PTX-loaded degradable cationic nanogel comprising of acetylated pullulan and low molecular weight polyethyleneimine. The cationic charge on the nanogel was restored by coating with HA, making it susceptible to degradation by the hyaluronidase in the tumor microenvironment. The HA-coated nano-chemotherapeutics showed enhanced tumoral permeation with a 2-fold increase in the amount of nanogel localized within the deep tissue regions when compared to non-HA systems. Zhang and Xu (2018) fabricated a bienzyme-responsive multifunctional envelope-type mesoporous silica nanoparticles in which the nanoparticles were first functionalized with HA for successful targeting to breast cancer cells and then exposing the system to hyaluronidase-induced intracellular release of chemotherapeutic. The outer layer of the nanoparticles contained a capping layer of gelatin, grafted via glutaraldehyde-mediated cross-linking. Further, in order to protect and prolong the systemic circulation of nanoparticles, they were pegylated. Post-administration, on exposure to the MMP-2 enzymes over-expressed by the cells, the gelatin was hydrolysed, de-shielding the PEG coating. This exposed the HA and led to hyaluronidase-mediated degradation of HA within the tumor microenvironment for successful drug delivery. In-order to explore the dual benefits of redox- and enzyme-responsiveness, Xu et al. (2015) developed DTX-loaded shrapnel nano delivery system. The nanocarrier system comprised of methoxy PEG-peptide-vitamin E succinate, a MMP-sensitive co-polymer, synthesized by conjugating PEG and vitamin E succinate using an enzyme-sensitive peptide. Further, drug-loaded PEG-disulfide-vitamin E succinate micelles were incorporated into PEG-peptide-vitamin E-based liposomes. This unique nanocarrier exhibited shrapnel structure with average particle size 113.3 ± 2.7 nm. These dual-responsive nano-chemotherapeutics were capable of inhibiting the metastasis and growth of breast cancer simultaneously.

## Conclusions

Tumor microenvironment has been implicated in cancer growth and metastasis. With growing understanding of tumor, it is established that tumors grow in highly heterogeneous and complex microenvironment consisting of the ECM components, immune cells, vasculature, TAMs, and CAFs. Recent advancements pinpoints that the alteration of tumor microenvironment and its abnormal composition is an important strategy in curtailing cancer progression, invasion, and metastasis. With the advent of nanotechnology in drug delivery arena, newer approaches to tackle growing menace of cancer have evolved. However, the complexity of tumor microenvironment has shown to play a crucial, yet controversial role in regulating deeper tumoral penetration of nano-chemotherapeutics and subsequently, their biological effects. Tackling this obstacle, strategies have been designed using nanotechnology to overcome acquired resistance induced by tumor milieu either by targeting tumor vasculature or by altering the stromal properties or by exploiting the tumor chemical microenvironment.

Hence, nano-chemotherapeutics can alter the tumoral drug delivery by inducing pertubations in the tumor microenvironment. Thus, nanotechnology offers a versatile tool by enabling delivery of either single or combination of chemotherapeutics alongwith multiple targeting ligands to specifically target overexpressed receptors or enzymes or reductive environment, a common feature of tumor microenvironment. This approach confers target specificity thereby providing efficacious therapy with minimal adverse off-target effects. Besides this, the growing understanding of targeting tumor microenvironment using nanotools is paving way for designing combined strategy of therapeutics and diagnostics viz. nanotheranostics. With growing number of clinical trials on nanotherapy, different strategies combining nano-chemotherapeutics with radiotherapy and other allied therapies will translate into successful strategy for overcoming drug resistance.

Overall, it can be stated that nano-chemotherapeutics does hold promise in the early stages of cancer, as these highly multifunctionalized nanocarriers ensure delivery of chemotherapeutics either by exploiting EPR effect or tumor microenvironment. However, there still lies a challenge in translating this success from bench to bedside. As from a commercialization perspective, there is still a need for an in-depth stability and nanotoxicology studies to ensure regulatory compliance.

## Author contributions

CF and DS devised the outline, drafted as well as revised the manuscript. MY reviewed the manuscript, contributed to the Figure 3, summarized the review abstract and conclusion. All authors have seen and agreed on the final version of the manuscript.

### Conflict of interest statement

The authors declare that the research was conducted in the absence of any commercial or financial relationships that could be construed as a potential conflict of interest.
